# A Framework for Assessing the Opportunity for Advanced Research, Development, and Regulatory Approval of Medical Countermeasures: A Component of BARDA's Emerging Infectious Diseases Strategy

**DOI:** 10.1093/infdis/jiaf486

**Published:** 2025-10-09

**Authors:** Richard C White, Rachael G Lewis, James D Little, Brenda L Fredericksen, Carol L Sabourin, M Chelsea Lane, Shannon G Loelius, Kimberly A Hofmeyer, Matthew Steele, Xiaomi Tong, Robert A Johnson

**Affiliations:** Center for Biomedical Advanced Research and Development Authority, Administration for Strategic Preparedness and Response, US Department of Health and Human Services, Washington, DC, USA; Booz Allen Hamilton, McLean, Virginia, United States of America, Supporting the Biomedical Advanced Research and Development Authority (BARDA), Administration for Strategic Preparedness and Response (ASPR), U.S. Department of Health and Human Services (HHS), Washington, DC, USA; Center for Biomedical Advanced Research and Development Authority, Administration for Strategic Preparedness and Response, US Department of Health and Human Services, Washington, DC, USA; Center for Biomedical Advanced Research and Development Authority, Administration for Strategic Preparedness and Response, US Department of Health and Human Services, Washington, DC, USA; Tunnell Government Services, Berwyn, Pennsylvania, United States of America, Supporting the Biomedical Advanced Research and Development Authority (BARDA), Administration for Strategic Preparedness and Response (ASPR), U.S. Department of Health and Human Services (HHS), Washington, DC, USA; Center for Biomedical Advanced Research and Development Authority, Administration for Strategic Preparedness and Response, US Department of Health and Human Services, Washington, DC, USA; Center for Biomedical Advanced Research and Development Authority, Administration for Strategic Preparedness and Response, US Department of Health and Human Services, Washington, DC, USA; Center for Biomedical Advanced Research and Development Authority, Administration for Strategic Preparedness and Response, US Department of Health and Human Services, Washington, DC, USA; Center for Biomedical Advanced Research and Development Authority, Administration for Strategic Preparedness and Response, US Department of Health and Human Services, Washington, DC, USA; Tunnell Government Services, Berwyn, Pennsylvania, United States of America, Supporting the Biomedical Advanced Research and Development Authority (BARDA), Administration for Strategic Preparedness and Response (ASPR), U.S. Department of Health and Human Services (HHS), Washington, DC, USA; Center for Biomedical Advanced Research and Development Authority, Administration for Strategic Preparedness and Response, US Department of Health and Human Services, Washington, DC, USA

**Keywords:** emerging viral pathogens, medical countermeasures, national health security, vaccine and therapeutic development

## Abstract

**Background:**

The advanced research, development, and regulatory approval of medical countermeasures (MCMs) for emerging pathogens remains critical to national health security. We created a conceptual framework to assess the feasibility of generating pivotal data to support FDA regulatory approval of vaccines and therapeutics against known pathogens. Our framework is intended to guide key portfolio decisions on developing MCMs for emerging viral pathogens.

**Methods:**

This framework draws on prior experience with MCM development programs, current FDA guidance, and insights from scientific, regulatory, and public health subject matter experts. We identified key requirements that impact the ability to generate pivotal data and assessed the likelihood of meeting these requirements based on current epidemiology, technical capabilities, and infrastructure. To demonstrate utility, we applied the framework to a subset of prioritized pathogens.

**Results:**

We identified eight factors central to assessing the feasibility of advanced development and FDA approval of vaccines and therapeutics. These factors were used to evaluate seven emerging pathogens to illustrate how the framework may inform investment decisions across disease contexts.

**Conclusions:**

This framework supports more efficient resource allocation by highlighting MCM candidates with the highest potential for FDA approval within existing regulatory paradigms. Given current conditions, vaccine development appears more feasible than therapeutics for the pathogens assessed, although regulatory pathways remain product- and context-specific. Close consultation with the FDA will be critical in defining appropriate regulatory strategies. This framework offers a structured, proactive approach to advance MCM development and strengthen national preparedness against emerging pathogens.

## Teaser

A conceptual framework to guide medical countermeasure development decisions and assess the feasibility of generating pivotal data to support FDA approval for vaccines and therapeutics against emerging viral pathogens.

The mission of the Center for Biomedical Advanced Research and Development Authority (BARDA) is to support the advanced development, regulatory approval, and procurement of medical countermeasures (MCMs) that address chemical, biological, radiological, and nuclear (CBRN) threats, pandemic influenza, and emerging infectious diseases [1]. BARDA primarily focuses on late-stage product development of vaccines, therapeutics, and diagnostic tools by supporting pivotal clinical trials, nonclinical studies, and Current Good Manufacturing Practice efforts to provide the US Food and Drug Administration (FDA) with important data required to potentially secure FDA regulatory approval. Since its inception in 2006, BARDA has built a diverse portfolio of MCMs, ultimately resulting in 100+ FDA approvals, licensures, or clearances to date [2].

Emerging viral pathogens continue to pose a threat to national security, with known pathogens causing more frequent outbreaks and/or expanding their geographical range [3]. The substantial number of known pathogens, in addition to budgetary constraints, make it infeasible to develop MCMs for every possible threat. To this end, BARDA has developed a comprehensive strategy to improve preparedness and response capabilities for emerging viral pathogens and guide future efforts in protecting the nation against evolving biothreats [4]. BARDA has prioritized flexible capabilities, including platform technologies, to support national health security, while investing in products through to FDA approval and stockpiling priority products for known threats. Overall, BARDA's efforts support a more efficient public health response to emerging viral pathogen outbreaks.

In this report, we review the considerations for generating pivotal clinical and nonclinical data to support FDA regulatory approval through traditional and alternative pathways for vaccine and therapeutic MCM product development. Although diagnostic and device development is beyond the scope of this report, their importance should not be understated as they complement many aspects of MCM development discussed herein. While this report specifically focuses on assessing available regulatory pathways and feasibility to generate required efficacy data for MCMs for emerging viral pathogens, we acknowledge that other critical factors, such as manufacturability, stability, and safety also influence overall product developability and are addressed at other points in BARDA's portfolio management process. Given BARDA's focus on national health security, this paper concentrates only on requirements needed to obtain product approval from FDA and does not consider approval requirements from other stringent regulatory authorities. The selected considerations are critical to ensuring the efficient allocation of resources and timely availability of MCMs during public health emergencies.

Although every pathway requires clinical demonstration of a robust safety profile in humans, each maintains a different approach to demonstrating pivotal evidence of product efficacy. The traditional regulatory pathway relies on data from adequate and well-controlled Phase 3 clinical trials to demonstrate efficacy needed for product approval. Accelerated Approval is an alternative pathway that allows for products that treat serious conditions and fulfill an unmet medical need to be approved based on a surrogate endpoint [5]. The Animal Rule is another alternative regulatory pathway for serious or life-threatening conditions caused by exposure to lethal or permanently disabling toxic CBRN substances. For this pathway to be considered, human efficacy trials must be neither feasible nor ethical, and approval is not possible under other pathways. The Animal Rule pathway, like Accelerated Approval, requires post-marketing studies that verify and describe the clinical benefit and assess product safety, when used as indicated and when such studies are feasible and ethical (e.g., during a response event) [6].

As BARDA structures its portfolio for emerging viral pathogens, having a framework to systematically assess the suitability of supporting MCM candidates with potential to receive FDA approval through specific regulatory pathways is critical. Ultimately, the regulatory strategy will be determined in consultation with FDA, therefore early engagement between BARDA, sponsors, and FDA will be critical in guiding efforts to support advanced development of product- and threat-specific MCMs. Leveraging a framework that evaluates key factors in MCM research and development will provide a valuable tool to inform BARDA's portfolio management approach and support data-driven decisions. Overall, this framework will promote efficiency in the advancement of MCMs from the research and development stages through to regulatory approval.

## METHODS

To develop a framework for assessing the feasibility of generating data to support regulatory approval for MCMs, we reviewed FDA guidance, in addition to peer-reviewed and gray literature, to identify the data and circumstances most conducive to potential approval from a regulatory authority [7–9]. We also consulted a team of subject-matter experts at BARDA and leveraged their prior experience with MCM development programs to guide our efforts, given the integral role these experts have played in 100+ FDA product licenses, approvals, and clearances for BARDA-supported products [2]. Aligning our framework with regulatory guidance and practical applications, we prioritized factors that could be systematically evaluated using publicly available information coupled with subject matter expertise to ensure the framework could be applied to diverse pathogens and scenarios. Factors were excluded if they could not be applied universally. Additionally, we focused exclusively on vaccine and therapeutic clinical/nonclinical product development. For therapeutics, we did not consider prophylactics. Selected factors are presented in Table 1, along with rationales for inclusion and rating criteria.

**Table 1.** Factors for Assessing Feasibility of Regulatory Pathways for Medical Countermeasures

Regulatory Pathway	Factor	Description and Levels	Rationale for Inclusion
Key considerations for traditional pathway	Spatiotemporal disease predictability and incidence	*The location of and number of confirmed cases over a specified period of time.* **Low—**sporadic/unpredictable outbreaks with low case counts. **Medium—**localized hot spots, and/or predominantly asymptomatic cases. **High—**hyperendemicity, ongoing/predictable transmission, high case counts.	Higher case numbers with evidence of spatial correlation might make traditional efficacy trials feasible, while low case counts or spatially unrelated cases may pose additional challenges to conducting a traditional efficacy study.
	Research infrastructure and capacity	*The capacity for MCM research activities in areas with cases. Contributing variables include, but are not limited to: laboratory capacity, health system supply chains, epidemiology workforce, healthcare access, clinical trial capacity, and others.* **Low**—Extreme limitations on healthcare access, health systems processes, and clinical trial capacity. **Medium**—Some evidence of healthcare/research capacity, with substantial limitations identified in key capacities. **High**—Strong clinical research infrastructure, healthcare access, stable supply chains, etc.	Limited in-country research capacity might constrain the ability to conduct large-scale Phase 3 trials and pursue traditional regulatory approval.
	Effective surveillance systems	*Evidence of pathogen-specific surveillance systems, including diagnostic capabilities, in disease-prone areas.* **Low**—Limited evidence of successful or robust surveillance systems. **Medium**—Some evidence of modest surveillance systems that may require adaptation, potential delays in accurate case identification. **High**—Comprehensive and routine surveillance systems with well-established and timely case identification processes and diagnostic capabilities.	Limitations or delays in viral detection and confirmation of the causative agent during a suspected outbreak could hinder timely treatment and delay initiation of trials for pathogen-specific vaccines or therapeutics.
	Therapeutic treatment window	*Evaluates the feasibility of testing antivirals in an interventional trial based on the therapeutic treatment window, ie, the time between initial viral detection and peak viral activity.* **Short**—Narrow treatment window, with viral clearance or severe disease occurring rapidly after detection. **Moderate**—Treatment window allows some feasibility for evaluating antivirals, but patient variability or rapid disease progression may introduce challenges in trial design and endpoint selection. **Long**—Treatment window is extended, providing a broader opportunity for enrollment and evaluation of therapeutic efficacy.	A longer treatment window allows for greater flexibility in trial design, recruitment, and outcome assessment, whereas a short window limits the opportunity for intervention and increases challenges in patient stratification and controlling for confounders.
…			
Key Considerations for Alternative Approval Pathways	Availability of immune markers for vaccine development	*Level of consensus in the scientific community on immune markers that may predict clinical efficacy for vaccines.* **Low**—No proposed immune markers and/or mechanisms of protection largely unknown. **Medium**—Proposed markers with general consensus on mechanisms of protection and supportive evidence (eg, passive transfer study). **High**—Validated and widely accepted markers; widely available international reference standards and other standardized reagents/protocols for harmonization.	Strong consensus may favor Accelerated Approval based on immunological markers.
	Well-characterized animal models	*Availability/characterization level of animal models that reliably mimic human disease progression and outcomes, enabling evaluation of MCM efficacy in the absence of human clinical data.* **Low—**Host susceptibility in relevant species and relevant route(s) of administration are known. **Medium—**Model defining natural history studies characterizing time to onset, disease progression, and manifestations have been conducted. **High—**Well-characterized model(s) reflecting key characteristics of the human disease are available at facilities with adequate quality management systems and regulatory concurrence for the context of use has been obtained.	Marketing approval under the Accelerated pathway may require the use of animal models to establish surrogates of protection or immune markers, while approval under the Animal Rule is based upon adequate and well-controlled animal efficacy studies. Therefore, the selection of an animal model should be based on the adequacy of the model to reflect key characteristics of the human disease or condition and its suitability regarding the investigational product. Regulatory concurrence is critical to the selection of an animal model for the proposed context of use.
	Characterization of challenge agent	*Extent to which the pathogen strain used in animal studies has been defined, including its origin, genetic makeup, and virulence, ensuring consistency and relevance to human infection.* **Low—**Strain selection: pathogen strain is same as the etiologic agent that causes the human disease or condition and applicable pathology seen in animal models. **Medium—**Stocks are generally available and can be used for product development, but the emergence of a novel strain will require the production and characterization of a new stock; general regulatory acceptance for model characterization. **High—**Well-characterized, centralized stocks available for funded research use (e.g., Certificate of Analysis, passage history).	Essential element of an animal model for alternative approval pathways. The characteristics of the specific etiologic or challenge agent that influence the disease or condition under study include its pathophysiological mechanisms of toxicity or virulence, the route of exposure, and the dose and quantification of exposure.
	Viral assays	*Denotes the presence of validated laboratory assays capable of accurately detecting, quantifying, and characterizing the virus in animal studies.* *Note that this excludes immunoassays (these are virus detection only).* **Low**—Assay is developed. **Medium**—Assay is optimized/well-characterized. **High**—Assay is qualified or validated according to regulatory guidance.	Robust viral assays are critical for measuring pathogen levels and assessment in adequate and well-controlled animal bridging and efficacy studies.

While each key consideration is aligned with the most relevant regulatory pathway (ie, Traditional, Alternative), all considerations may be relevant across multiple pathways and are evaluated collectively when determining overall regulatory feasibility.

The framework methodology centers around distinguishing the key pieces of evidence that are needed to support potential product approval and identifying factors aligned with pivotal data requirements for each pathway. Generating evidence to support the traditional regulatory pathway primarily involves a pivotal clinical efficacy trial where MCM safety and efficacy is directly demonstrated in an adequate and well-controlled study providing robust evidence of clinical benefit [10]. Data to support Accelerated Approval relies on surrogate efficacy endpoints—such as immune responses or biomarkers—that are thought to be reasonably likely to predict clinical benefit but are not themselves a measure of clinical benefit. This pathway requires post-approval confirmatory studies demonstrating clinical benefit and safety [5]. Product licensure under the Animal Rule requires human safety and pharmacokinetic data (including establishing a link between animal protection and human protection through immune markers for vaccines) as demonstrated in clinical trials. Animal Rule requires adequate and well-controlled challenge/protection studies in animal models to support that the MCM is “reasonably likely to produce clinical benefit in humans.” [11] Animal Rule approval is subject to three requirements: (1) post-marketing studies that verify and describe the clinical benefit and assess safety (when the product is used as indicated and when such studies are feasible and ethical); (2) approval restrictions to ensure safe use, if needed; and (3) special labeling to patient recipients that explains that efficacy was demonstrated in animal models alone [6]. These key considerations for the traditional and alternative regulatory pathways guided our methodology in identifying corresponding factors and assessing results for prioritized pathogens.

**Figure 1.**
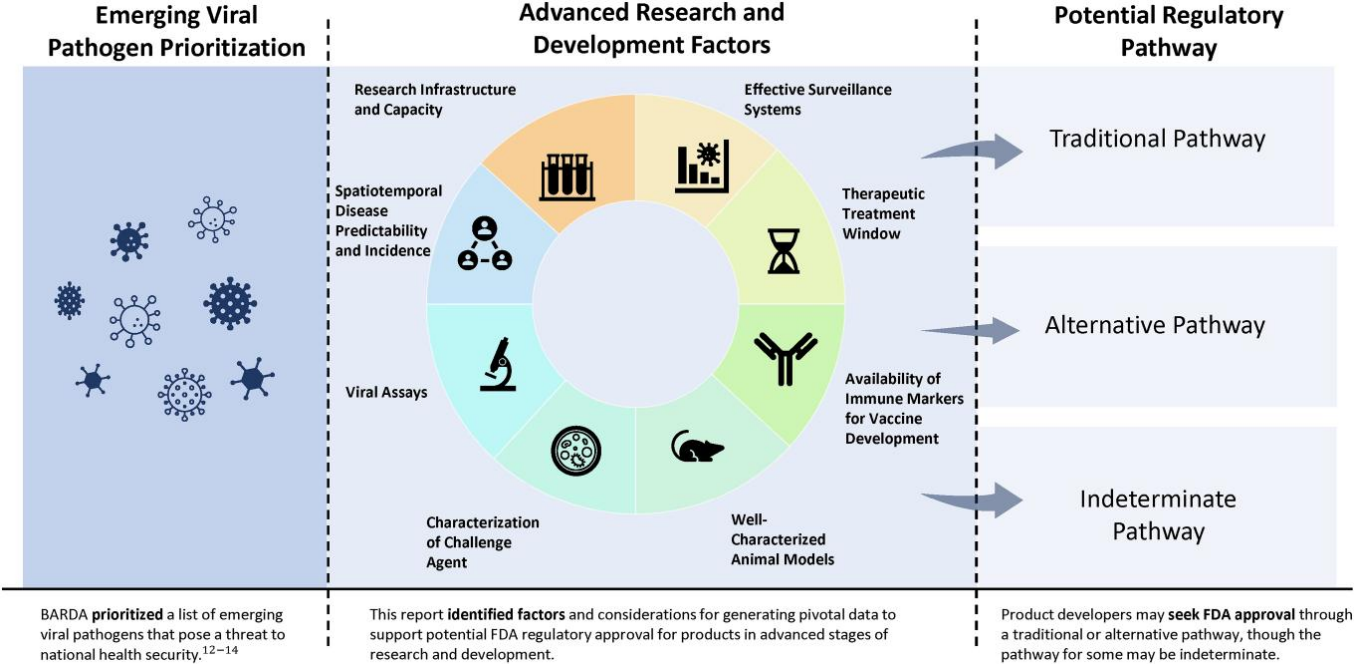
Assessing the opportunity for advanced development and regulatory approval of medical countermeasures. This figure displays the process of identifying opportunities for advanced research and development of vaccines and therapeutics seeking potential US Food and Drug Administration (FDA) regulatory approval. This schematic is a component of BARDA's emerging viral pathogen strategy.

**Figure 2.**
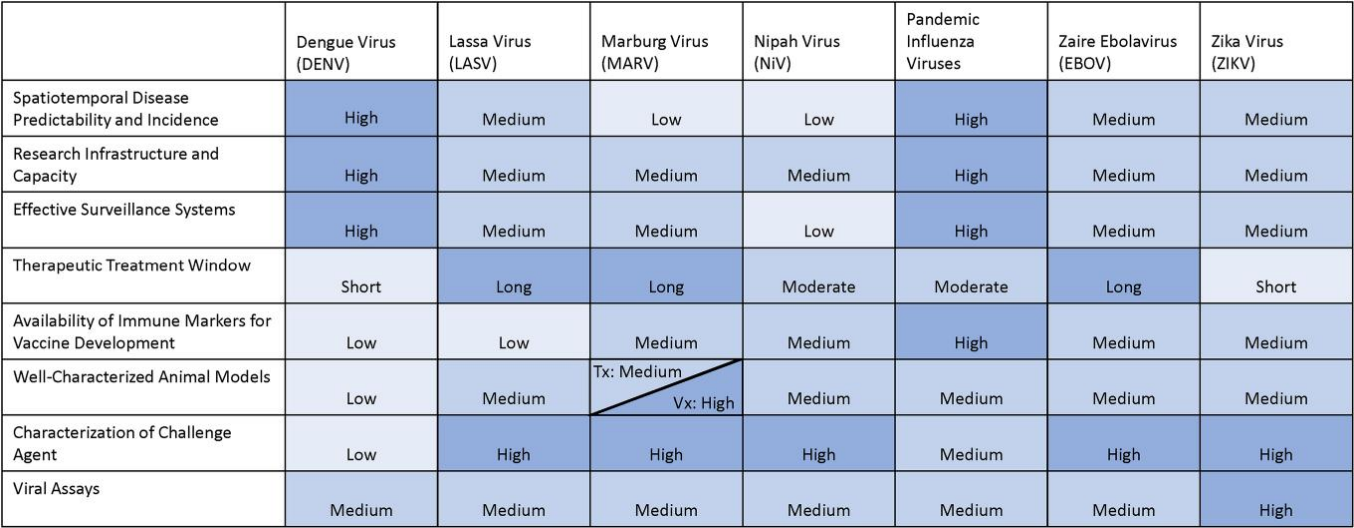
A heat map of results for prioritized emerging viral pathogens. Shading is correlated with factor-specific ratings; pathogens with factor-specific results of “high” are displayed with a darker shade than those that are “medium” or “low.” If the results of a specific factor differ between vaccines and therapeutics for a specific pathogen, the respective cell is displayed with a diagonal break. For purposes of this feasibility assessment, pandemic influenza was evaluated based on available data for seasonal influenza infections. Historically, most vaccines and therapeutics that have been approved or deployed for pandemic influenza strains have been supported by efficacy data derived from seasonal influenza. Abbreviations: Tx, therapeutic; Vx, vaccine.

Finally, to demonstrate the utility of the framework described herein, we evaluated the factors against seven selected emerging viral pathogens previously assessed by BARDA [12]. These viral pathogens and their respective pathogen families have been identified as high-priority threats by the Public Health Emergency Medical Countermeasures Enterprise (PHEMCE) and other public health agencies [13–15]. As described in Supplementary Data, we examined myriad data sources to determine factor-specific levels for each viral pathogen. A schematic outlining the relationship between the prioritized pathogens, framework factors, and regulatory pathways described in this report is illustrated in Figure 1. Results are displayed in Figure 2.

## RESULTS

### Key Factors for Traditional Approval Pathway

#### Spatiotemporal disease predictability and incidence

One of the greatest challenges in assessing MCM efficacy against many of these viral threats is their spatiotemporal variability in incidence [16]. To illustrate, emerging viral pathogens such as dengue virus (DENV) are hyperendemic in many regions around the world and produce hundreds of thousands of spatially and temporally related cases each year, which indicates that supporting a traditional efficacy study may be feasible. On the other hand, outbreaks of other pathogens such as Nipah virus (NiV) do not often produce enough cases to sufficiently power a traditional efficacy study [17, 18]. Therefore without a change in epidemiology for these pathogens, and regardless of their status with other factors in the framework, it may be more feasible to support research efforts to generate data aligned with an alternative pathway instead. This factor will be useful to identify the types of data for each prioritized pathogen that would best support potential regulatory approval through a traditional or alternative pathway.

#### Research Infrastructure and Capacity

In addition to targeted assessments of clinical research capacity, this factor was guided by previously published capacity assessments such as the ESSENCE on Health Research Initiative, the International Health Regulations States Parties Self-Assessment Annual Reporting Tool (SPAR), and the Global Health Security (GHS) Index [19–21]. Capabilities assessed within this factor include (but are not limited to): healthcare access, clinical trial capacity, lab systems strength, and epidemiology workforce. To illustrate, the ESSENCE, GHS, and SPAR indices report a high capacity for clinical trial research in countries where there is ongoing transmission of DENV. Furthermore, FDA's approval of a DENV vaccine, which was supported by pivotal efficacy trials conducted in dengue-endemic countries [22], demonstrates that the infrastructure necessary to carry out large-scale clinical trials in these settings exists-reinforcing the feasibility of future MCM development and approval for DENV. Overall, this factor supports the potential for future research endeavors that could enable regulatory approval for MCMs for specific emerging viral pathogens.

#### Effective Surveillance Systems

Because traditional therapeutic clinical trials depend on the enrollment of suspected or confirmed cases, the opportunity for regulatory approval of a treatment therapeutic is greatly enhanced by robust and accurate surveillance systems, including diagnostic capabilities. Likewise, reactive vaccine trials for outbreak response also rely on positive identification of an index case for trial initiation. For instance, even though filovirus cases may be sporadic and unpredictable in nature, effective surveillance and diagnostic capabilities may enhance rapid response efforts and enable a ring vaccination approach to be used once an index case is identified [3]. A flexible trial approach can be subsequently leveraged to demonstrate vaccine efficacy in field trials, as exemplified by the approval of ERVEBO for Ebola virus disease (EVD) utilizing a ring vaccination approach [3, 23]. Hence, robust surveillance and diagnostic systems are vital for multiple aspects of MCM development.

#### Therapeutic Treatment Window

Conducting traditional efficacy trials for emerging viral pathogen therapeutics presents significant challenges due to restrictions in the therapeutic window and limitations in timely diagnosis and treatment initiation. We define the treatment window for antiviral therapeutics as the period from initial viral detection to peak viral activity (when antivirals are most likely to demonstrate measurable clinical benefit). For instance, the critical administration timeline for influenza antivirals, such as oseltamivir, is 48 hours after symptom onset given that peak viral load occurs within 1–2 days [24, 25]. For other emerging viral pathogens, such as filoviruses (eg, EBOV, MARV), the treatment window may be wider, as there is a longer duration of viral activity and peak viral load often occurs between days 5–7 [26, 27]. Even still, treatment effectiveness is strengthened by early intervention, which highlights the need for and use of highly sensitive, point-of-need diagnostic capabilities to identify and initiate treatment as early as possible [28–30]. Beyond this treatment window, the disease course may progress to a post-viral syndrome phase, in which symptoms are driven more by immune or inflammatory responses rather than by active viral replication. For some pathogens (eg, dengue virus, Zika virus), severe disease manifests after peak viral load or even after viral clearance, highlighting the need for additional therapeutic strategies, such as targeting immune dysregulation [31, 32]. These strategies may include testing syndrome-based therapeutics or combination regimens. While such strategies were beyond the scope of this assessment, BARDA continues to review such approaches on a case-by-case basis [24].

### Key Factors for Alternative Approval Pathways

#### Availability of immune markers for vaccine development

Vaccine licensure through an alternative pathway may be feasible if the traditional pathway is not possible and data exist to support a well-established immune marker to support licensure through Accelerated Approval or Animal Rule. For example, FDA has accepted hemagglutination inhibition (HAI) titers as a surrogate endpoint for pandemic influenza vaccine candidates [33]. Vaccine candidates for pandemic influenza viruses that meet the proposed surrogate endpoint fit the requirements needed to seek Accelerated Approval, given that an adequate and well-controlled clinical trial to verify and describe clinical benefit is feasible following licensure during a public health emergency. More recently, FDA granted Accelerated Approval for a Chikungunya vaccine based on a surrogate endpoint (ie, CHIKV neutralizing antibody titer) [34], providing a compelling example of how immune markers can facilitate approval for vaccines targeting emerging viral pathogens. Moreover, the Chikungunya vaccine experience may serve as a model for other arbovirus vaccines, such as those targeting dengue virus and Zika virus, given the similarities in modes of transmission, outbreak unpredictability, and initial clinical presentations [33, 35]. Overall, FDA will evaluate proposed immune markers for other emerging viral pathogen vaccines on a case-by-case basis.

#### Characterization of Challenge Agent

Central to the development of a well-characterized animal model is the availability of an appropriate and well-characterized challenge agent. We assessed the “readiness level” (ie, how well current tools and data support efficacy evaluation) of available challenge agents for the prioritized pathogens. Our assessment was based on the following criteria: (1) the challenge agent in animals is the same etiologic agent in humans; (2) a well-characterized, low-passage challenge agent with well-documented provenance and passage history is available in an accessible repository; (3) the challenge agent was associated with an outbreak of human disease; and (4) dose, route of exposure and the pathophysiological mechanisms of virulence have been determined. The MARV Angola challenge stock represents an example that met all the criteria listed above and thereby received a “high” readiness level. The MARV isolate was isolated from a fatal human case in Angola during the 2005 outbreak and low-passage challenge stocks are available through special request from Biodefense and Emerging Infections Research Resources Repository (BEI Resources) [36]. The provenance and passage history for these stocks is well documented and the isolate has been demonstrated to be uniformly lethal in nonhuman primates (NHPs).

#### Viral Assays

Reliable quantification of the challenge agent is important for both establishment of an animal model and assessing the efficacy of candidate MCMs. We did not include immunological assays in this assessment because these types of assays are, by necessity, product dependent. We instead focused on status of product agnostic virological assays to quantify live virus and viral genomic material. In general, assays used to assess viral loads in published studies were considered developed and at a low responsiveness level. Assays were only considered to be optimized/well-characterized (medium responsiveness level) or qualified/validated (high responsiveness level) if BARDA had access to a report or publication describing optimization, qualification, or validation process for assays to quantify both live virus and viral genomic material. For example, although the plaque assay for Ebola Zaire has been validated, Ebola Zaire is only considered to have an overall assay readiness status of medium because the RT-qPCR assay has only been optimized for NHP samples [37].

#### Well-Characterized Animal Models

Readiness level of each pathogen's animal model was assessed based on: (1) the availability of natural history studies documenting the pathogenesis of the viral agent and supporting the relevance of the animal model to human disease; (2) the availability of the model in one or more laboratories with quality systems required to conduct adequate and well-controlled efficacy studies; and (3) the model has been received favorably by the relevant regulatory authority for the proposed context of use. The NHP model for MARV was determined to be at a high readiness level for assessing the efficacy of vaccine candidates because there is an extensive dataset demonstrating the pathogenesis and relevance of the MARV Angola strain in NHPs [38]. Although a product agnostic model may be available and endorsed by regulatory authorities, sponsors of candidate MCMs must “provide justification of the suitability of each model based on the investigational drug's mechanism of action, dosage form, and route of administration, and the method proposed to extrapolate from the animal data to select a dose and regimen in humans.” [8] Additionally, the FDA Center for Drug Evaluation and Research (CDER) has not endorsed a MARV NHP model for conduct of adequate and well-controlled efficacy studies for therapeutic product. Thus, the NHP model was considered to be at a medium responsiveness level for therapeutics candidates but a high level for vaccine candidates.

## DISCUSSION

This study presents a structured framework for assessing the feasibility of generating pivotal data to support FDA regulatory approval of MCMs for emerging viral pathogens. To validate the framework's utility, we applied it to multiple emerging viral pathogens that pose a risk to national health security [12–15]. We demonstrated how BARDA may approach specific pathogens, leveraging available knowledge to assess the likelihood of regulatory approval and inform portfolio decisions. By systematically evaluating the feasibility of supporting MCMs through to approval, this tool supports prioritization efforts and ensures that BARDA's limited financial resources are directed toward MCM candidates with the highest potential for success within existing regulatory paradigms. Likewise, this tool can highlight gaps where BARDA may be able to support additional data/improvement efforts should a need arise for an MCM against a specific viral pathogen.

Our assessment indicates that, for some assessed pathogens, it is currently feasible to generate data supporting vaccine licensure. In instances where sufficient clinical data and immune markers can be established for vaccine candidates, data suggest that the traditional efficacy and/or Accelerated Approval pathways may be viable. However, the regulatory pathway for therapeutics remains less certain. In some cases, approval may be possible under the Animal Rule, although this pathway is intended as a “last resort” and may not be feasible given the current disease context. Given available technologies, developing therapeutic products for many of BARDA's prioritized viral pathogens may not be feasible. Additional research, improved diagnostics and surveillance capabilities, and a shift in epidemiology may be necessary to achieve regulatory approval for therapeutic candidates.

The results of this report do not guarantee that all current and emerging threats will meet the requirements needed to seek FDA approval. Provided that some emerging viral diseases may not have products in advanced development stages currently, it may remain unclear which regulatory pathway is most feasible to pursue. Therefore, the approach to approval may be indeterminate due to the current disease context, available technologies, or resource constraints. Early engagement with FDA is critical to identify the most suitable regulatory pathway and ensure alignment with agency expectations.

The success of MCM development will be strengthened not only by BARDA's investments but also through coordination with key partners including product developers, academia, and funding bodies such as Gavi, the Gates Foundation, and the Coalition for Epidemic Preparedness Innovations (CEPI). These partnerships are essential for advancing scientific understanding of disease models, elucidating mechanisms of protection, enhancing global surveillance capabilities, and expanding research infrastructure. Our framework will help identify pathogen-specific capacities where additional research and support are needed, thereby reducing the duplication of efforts by BARDA and its partners while promoting an efficient approach to supporting MCMs through to FDA approval. A coordinated, multi-stakeholder approach will be critical to overcoming existing barriers to MCM development and ensuring that promising candidates can progress efficiently through the regulatory process [39].

This framework is not without limitation. First, it represents a snapshot in time and should be periodically updated as new information emerges, such as the occurrence of additional human cases, the development of new animal models, or the evolution of regulatory guidance. Any proposed regulatory strategy must involve early and frequent engagement with FDA to ensure alignment with agency expectations. Furthermore, each pathway evaluated in this study presents inherent challenges and limitations. Traditional efficacy studies rely on a set of critical assumptions not addressed here, including the availability of investigational product in-country and pre-approved protocols across multiple jurisdictions. Additionally, despite strong research in potential immune correlates for vaccine development, there is limited regulatory precedent for therapeutic products. Multiple practical challenges arise because biomarkers and surrogate endpoints for antiviral therapeutics are typically measured in infected individuals. For instance, there may be too few infections to generate sufficient data to support Accelerated Approval. Even with sufficient cases, the acute nature of the viral illness may necessitate the demonstration of direct clinical benefit (e.g., survival) rather than relying solely on a surrogate. As a result, the Accelerated Approval pathway is likely of greater utility to vaccines than therapeutics for acute viral illnesses [9]. Additionally, Animal Rule approval depends on the availability of well-characterized animal models. This may present a challenge for pathogens that are not associated with a high rate of human mortality, or those that do not sufficiently recapitulate the pathology and severity of human disease in animal models thereby complicating the ability to demonstrate meaningful clinical endpoints. As BARDA looks to the future of advanced development of MCMs for emerging pathogens, this framework will be an important tool to help prioritize investments in vaccines and therapeutics that are most likely to reach FDA approval, ultimately contributing to national and global health security. Through continued refinement, strategic partnerships, and proactive engagement with regulatory authorities, BARDA remains committed to strengthening preparedness and response efforts against threats to national health security.

## Supplementary Material

jiaf486_Supplementary_Data

## Supplementary Data

Supplementary materials are available at *The Journal of Infectious Diseases* online (http://jid.oxfordjournals.org/). Supplementary materials consist of data provided by the author that are published to benefit the reader. The posted materials are not copyedited. The contents of all supplementary data are the sole responsibility of the authors. Questions or messages regarding errors should be addressed to the author.

## References

[1] Center for the Biomedical Advanced Research and Development Authority . Available at: https://aspr.hhs.gov/AboutASPR/ProgramOffices/BARDA/Pages/default.aspx. Accessed 14 April 2025.

[2] Biomedical Advanced Research and Development Authority (BARDA) . FDA Approvals, Licensures & Clearances for BARDA Supported Products. Available at: https://medicalcountermeasures.gov/barda/fdaapprovals. Accessed 14 April 2025.

[3] Parish LA, Stavale EJ, Houchens CR, Wolfe DN. Developing vaccines to improve preparedness for filovirus outbreaks: the perspective of the USA biomedical advanced research and development authority (BARDA). Vaccines 2023; 11:1120. 37376509 10.3390/vaccines11061120 PMC10301178

[4] Johnson RA, Barnhart TM, Disbrow GL. Building a fast response capability for emerging infectious diseases within the biomedical advanced research and development authority. Health Security 2025; 23(1):55–61. 39812164 10.1089/hs.2024.0074

[5] U.S. Food and Drug Administration (FDA) . Accelerated Approval Program. Available at: https://www.fda.gov/drugs/nda-and-bla-approvals/accelerated-approval-program. Accessed 16 December 2024.

[6] U.S. Food and Drug Administration (FDA) . New Drug and Biological Drug Products; Evidence Needed to Demonstrate Effectiveness of New Drugs When Human Efficacy Studies Are Not Ethical or Feasible. Available at: https://www.govinfo.gov/content/pkg/FR-2002-05-31/pdf/02-13583.pdf. Accessed 16 December 2024. 12049094

[7] U.S. Food and Drug Administration (FDA) . Guidance for Industry: General Principles for the Development of Vaccines to Protect Against Global Infectious Diseases. 2011. Available at: https://www.fda.gov/files/vaccines%2C%20blood%20%26%20biologics/published/Guidance-for-Industry--General-Principles-for-the-Development-of-Vaccines-to-Protect-Against-Global-Infectious-Diseases.pdf. Accessed 16 December 2024.

[8] US Food and Drug Administration (FDA) . Product Development Under the Animal Rule Guidance for Industry. Available at: https://www.fda.gov/regulatory-information/search-fda-guidance-documents/product-development-under-animal-rule. Accessed 16 December 2024.

[9] US Food and Drug Administration (FDA) . Guidance for Industry Expedited Programs for Serious Conditions—Drugs and Biologics Available at: https://www.fda.gov/files/drugs/published/Expedited-Programs-for-Serious-Conditions-Drugs-and-Biologics.pdf. Accessed 18 December 2024.

[10] US Food and Drug Administration (FDA) . Regulatory Perspective on Development of Preventive Vaccines for Global Infectious Diseases. Available at: https://www.fda.gov/media/134489/download?attachment. Accessed 16 December 2024.

[11] Beasley DWC, Brasel TL, Comer JE. First vaccine approval under the FDA animal rule. NPJ Vaccines 2016; 1:16013. 29263855 10.1038/npjvaccines.2016.13 PMC5707879

[12] White RC, Kovacs G, Chandran S, et al A framework for assessing viral pathogens: a key element of the BARDA emerging infectious diseases strategy. Health Security 2025; 23(1): 47–54. 39882823 10.1089/hs.2024.0076

[13] Administration for Strategic Preparedness and Response ASPR. PHEMCE Priority Threats. Available at: https://aspr.hhs.gov/PHEMCE/Pages/Priority-Threats.aspx. Accessed 2 May 2025

[14] NIAID Biodefense Pathogens . Available at: https://www.niaid.nih.gov/research/niaid-biodefense-pathogens#d. Accessed May 2 2025.

[15] Cassetti MC, Pierson TC, Patterson LJ, et al Prototype pathogen approach for vaccine and monoclonal antibody development: a critical component of the NIAID plan for pandemic preparedness. J Infect Dis 2023; 227:1433–41. 35876700 10.1093/infdis/jiac296 PMC9384504

[16] Points of Considerations and Key Principles. Geneva: World Health Organization; 2019. Available at: https://www.who.int/docs/default-source/blue-print/working-group-for-vaccine-evaluation-(4th-consultation)/ap1-guidelines-online-consultation.pdf. Accessed 10 January 2025

[17] Mohapatra P, Nazli Khatib M, Shabil M, et al Addressing the nipah virus threat: a call for global vigilance and coordinated action. Clin Infect Pract 2024; 24:100390.

[18] World Health Organization. Nipah Research and Development (R&D) Roadmap: October 2019 – Advanced Draft. Geneva: World Health Organization; 2019. Available at: https://www.who.int/blueprint/priority-diseases/key-action/Nipah_Roadmap_Advanced_Draft_Oct2019.pdf. Accessed 10 January 2025.

[19] Global Health Security Index . Available at: https://ghsindex.org/. Accessed 10 January 2024.

[20] Cruz JLC, Kilmarx PH. Analysis of country-level health research capacity for the ESSENCE on Health Research Initiative. Bethesda, MD: Fogarty International Center, National Institutes of Health, 2022.

[21] World Health Organization . International health regulations (2005): states parties self-assessment annual reporting tool. 2nd ed. Geneva: World Health Organization, 2021:4–51.

[22] US Food and Drug Administration (FDA) . Summary Basis for Regulatory Action. Available at: https://www.fda.gov/media/125157/download?attachment. Accessed 10 January 2025.

[23] Dean NE, Gsell P-S, Brookmeyer R, et al Design of vaccine efficacy trials during public health emergencies. Sci Transl Med 2019; 11:eaat0360. doi:10.1126/scitranslmed.aat0360 31270270 PMC6613811

[24] Jones JC, Yen H-L, Adams P, Armstrong K, Govorkova EA. Influenza antivirals and their role in pandemic preparedness. Antiviral Res 2023; 210:105499. 36567025 10.1016/j.antiviral.2022.105499 PMC9852030

[25] Li IW, Hung IF, To KK, et al The natural viral load profile of patients with pandemic 2009 influenza A(H1N1) and the effect of oseltamivir treatment. Chest 2010; 137:759–68. 20061398 10.1378/chest.09-3072 PMC7094292

[26] Jeremiah Matson M, Ricotta E, Feldmann F, et al Evaluation of viral load in patients with ebola virus disease in Liberia: a retrospective observational study. The Lancet Microbe 2022; 3:e533–42. 35617976 10.1016/S2666-5247(22)00065-9 PMC9254266

[27] Ogbaini-Emovon E, Akpede G, Okogbenin S, et al Virus load kinetics in Lassa fever patients treated with ribavirin: a retrospective cohort study from southern Nigeria. Open Forum Infect Dis 2024; 11:ofae575. 39450398 10.1093/ofid/ofae575 PMC11500659

[28] Kortepeter MG, Dierberg K, Shenoy ES, Cieslak TJ. Marburg virus disease: a summary for clinicians. Int J Infect Dis 2020; 99:233–42. 32758690 10.1016/j.ijid.2020.07.042 PMC7397931

[29] de L, Vega M-A, Caleo G, et al Ebola viral load at diagnosis associates with patient outcome and outbreak evolution. J Clin Invest 2015; 125:4421–8. 26551677 10.1172/JCI83162 PMC4665775

[30] Li J, Duan H-J, Chen H-Y, et al Age and Ebola viral load correlate with mortality and survival time in 288 Ebola virus disease patients. Int J Infect Dis 2016; 42:34–9. 26523640 10.1016/j.ijid.2015.10.021 PMC7110900

[31] Halai U-A, Nielsen-Saines K, Moreira ML, et al Maternal Zika virus disease severity, virus load, prior dengue antibodies, and their relationship to birth outcomes. Clin Infect Dis 2017; 65:877–83. 28535184 10.1093/cid/cix472 PMC5848222

[32] Khanam A, Gutiérrez-Barbosa H, Lyke KE, Chua JV. Immune-mediated pathogenesis in dengue virus infection. Viruses 2022; 14:2575. 36423184 10.3390/v14112575 PMC9699586

[33] US Food and Drug Administration (FDA) . Table of Surrogate Endpoints That Were the Basis of Drug Approval or Licensure. Available at: https://www.fda.gov/drugs/development-resources/table-surrogate-endpoints-were-basis-drug-approval-or-licensure. Accessed 10 January 2025.

[34] US Food and Drug Administration (FDA) . FDA Approves First Vaccine to Prevent Disease Caused by Chikungunya Virus. Available at: https://www.fda.gov/news-events/press-announcements/fda-approves-first-vaccine-prevent-disease-caused-chikungunya-virus. Accessed 10 January 2025.

[35] Gruber MF, Farizo KM, Pratt RD, et al Clinical development strategies and considerations for Zika vaccine licensure. J Infect Dis 2017; 216:S964–70. 29267913 10.1093/infdis/jix433 PMC5853280

[36] World Health Organization . Marburg haemorrhagic fever, Angola. Week Epidemiolog Rec 2005; 80:158–159. 15898301

[37] Shurtleff A, Bloomfield H, Mort S, et al Validation of the filovirus plaque assay for use in preclinical studies. Viruses 2016; 8:113. 27110807 10.3390/v8040113 PMC4848606

[38] Blair PW, Keshtkar-Jahromi M, Psoter KJ, et al Virulence of marburg virus Angola compared to mt. Elgon (musoke) in macaques: a pooled survival analysis. Viruses 2018; 10:658. 30469360 10.3390/v10110658 PMC6267608

[39] Swenson J, Disbrow G, Johnson RA. How global collaboration can improve the medical countermeasure life cycle for infectious disease outbreaks. J Infect Dis 2024; 230:e1–3. 39052706 10.1093/infdis/jiae017 PMC11272034

