# Supplementary material for: A Framework for Assessing the Opportunity for Advanced Research, Development, and Regulatory Approval of Medical Countermeasures: A Component of BARDA's Emerging Infectious Diseases Strategy
Source: J Infect Dis. 2025 Oct 9;233(1):e1–e10. doi: 10.1093/infdis/jiaf486 (PMC12811876; doi:10.1093/infdis/jiaf486)
Supplement: jiaf486_Supplementary_Data [file jiaf486_supplementary_data.docx]

Supplementary Table 1

|  | Dengue Virus (DENV) | Lassa  Virus (LASV) | Marburg Virus (MARV) | Nipah Virus (NiV) | Pandemic Influenza Viruses | Zaire Ebolavirus (EBOV) | Zika Virus (ZIKV) |
| --- | --- | --- | --- | --- | --- | --- | --- |
| Spatiotemporal Disease Predictability and Incidence | Hyperendemic, ongoing transmission [1-3] | Localized hot spots, typically a few hundred cases or less [4-7] | Sporadic outbreaks, small cluster size [8-10] | Small cluster size [11-13] | Localized hot spots <100 cases, primarily China, Egypt, North America, ongoing transmission of seasonal influenza [14-16] | Localized hot spots, typically a few hundred cases or less but some larger outbreaks [17, 18] | Ongoing transmission, diagnostic difficulties [19-22] |
| Research Infrastructure and Capacity | Ongoing clinical research [23-25] | Ongoing trials, infrastructure gaps identified [23-27] | Research capacity in some countries [23-25] | Research capacity in some countries, cases in rural areas ^[23-25]^ | Ongoing clinical research [23-25] | Research capacity in some countries ^[23-25]^ | Transmission in countries with moderate index scores ^[23-25]^ |
| Effective Surveillance Systems | Global surveillance systems [1, 28-31] | Some regional surveillance, diagnosis challenges [7, 32-34] | Some active regional surveillance, diagnostic challenging [9, 35] | Surveillance in hotspot areas, diagnostic challenges [11-13, 36] | Global surveillance systems [37-40] | Specialized surveillance programs, wider coverage needed [32, 33, 41-43] | Active surveillance programs, diagnostic challenging [28, 30, 31, 44, 45] |
| Therapeutic Treatment Window | Severe disease after peak viral load, early treatment may reduce viral load [46, 47] | Severe disease correlated with high viral load [48, 49] | Severe disease correlated with high viral load[50] | Tx window up to 5 days in some animal models, human data is lacking [51] | Virus replication peaks after 48 hours[52, 53] | Severe disease correlated with high viral load [54, 55] | No correlation between severity and viral load [56] |
| Availability of Immune Markers for Vaccine Development | Proposed immune markers not standard across serotypes[57-59] | Proposed immune markers differ between platforms[60-63] | Anti-MARV GP antibody proposed, no reference standard [64] | Immune markers proposed but not confirmed via passive transfer [65, 66] | HI titer is FDA-accepted correlate [67] | Anti-EBOV GP antibody proposed [68-71] | Immune markers proposed [72-74] |
| Well-Characterized Animal Models ^a^ | Low | Medium | ^Medium: Tx^  _High: Vx [75, 76]_ | Medium | Medium | Medium | High |
| Characterization of Challenge Agent ^a^ | Low | High | High | High | Medium | High | High |
| Viral Assays ^a^ | Medium | Medium | Medium | Medium | Medium | Medium | High |

**^a^** The ratings for some factors were informed by data generated by BARDA-funded product agnostic nonclinical studies that are not yet published and the institutional knowledge of BARDA subject-matter expertise. Therefore, direct references to peer-reviewed literature are not included. Abbreviation: HI, hemagglutination inhibition; GP, glycoprotein.

**Additional Information**

*In addition to the key factors identified in the framework above, additional data points may also inform BARDA portfolio decisions. While not essential for pursuing regulatory approval, these considerations can provide valuable context and will be factored into decision making when such data are available.*

|  | Dengue Virus (DENV) | Lassa  Virus (LASV) | Marburg Virus (MARV) | Nipah Virus (NiV) | Pandemic Influenza Viruses | Zaire Ebolavirus (EBOV) | Zika Virus (ZIKV) |
| --- | --- | --- | --- | --- | --- | --- | --- |
| U.S. Regulatory Precedent (Yes/No) | Yes  *Vx: Traditional [77]*  No*: Tx* | No | No | No | Yes  *Vx: Traditional and Accelerated Approval [78-80]*  *Tx: Traditional* [81, 82] | Yes  *Vx and Tx: Traditional [83-85]* | No |
| Prepositioned Clinical Trial Protocols | No | Yes  Tx[26, 86] | Yes  Vx[87, 88] and Tx[89] | No | Yes[80] | Yes  Vx and Tx [87, 89, 90] | No |
| Reference Standards | Yes[91] | Yes[92] | Yes[93] | Yes[94] | Yes[95, 96] | Yes[97] | Yes[98, 99] |

*Supplementary Table 1 displays a heat map of results for prioritized emerging viral pathogens with references included. Shading is correlated with factor specific ratings; pathogens with factor-specific results of “high” are displayed with a darker shade than those that are “medium” or “low.” If the results of a specific factor differ between vaccines and therapeutics for a specific pathogen, the respective cell is displayed with a diagonal break.*

References

1. WHO Global Dengue Dashboard Available at: <https://worldhealthorg.shinyapps.io/dengue_global/>. Accessed December 11, 2024.

2. Harapan H, Panta K, Michie A, et al. Hyperendemic Dengue and Possible Zika Circulation in the Westernmost Region of the Indonesian Archipelago. Viruses **2022**; 14:219.

3. Lim JT, Dickens BS, Tan KW, et al. Hyperendemicity associated with increased dengue burden. Journal of The Royal Society Interface **2021**; 18:20210565.

4. Greenky D, Knust B, Dziuban EJ. What Pediatricians Should Know About Lassa Virus. JAMA Pediatrics **2018**; 172:407.

5. Mylne AQN, Pigott DM, Longbottom J, et al. Mapping the zoonotic niche of Lassa fever in Africa. Transactions of The Royal Society of Tropical Medicine and Hygiene **2015**; 109:483-92.

6.  European Centre for Disease Prevention and Control. Annual Epidemiological Report 2019 – Lassa fever. Stockholm, **2021**.

7. Prevention ACfDCa. Lassa Fever Available at: <https://africacdc.org/disease/lassa-fever/>. Accessed January 10, 2025.

8. Qian GY, Jombart T, John Edmunds W. Assessing the feasibility of Phase 3 vaccine trials against Marburg Virus Disease: A modelling study. Vaccine: X **2023**; 14:100321.

9. Marzi A, Feldmann H. Marburg Virus Disease: Global Threat or Isolated Events? The Journal of Infectious Diseases **2023**; 228:103-5.

10. Organization WH. Marburg virus disease. Available at: <https://www.who.int/news-room/fact-sheets/detail/marburg-virus-disease>.

11. Khan S, Akbar SMF, Mahtab MA, et al. Twenty-five years of Nipah outbreaks in Southeast Asia: A persistent threat to global health. IJID Regions **2024**; 13:100434.

12. Mohapatra P, Nazli Khatib M, Shabil M, et al. Addressing the Nipah virus threat: A call for global vigilance and coordinated action. Clinical Infection in Practice **2024**; 24:100390.

13. Organization WH. Nipah Research and Development (R&D) Roadmap: , **2019**.

14. Fuller TL GM, Martin V, Cappelle J, Hosseini P, Njabo KY, et al. Predicting Hotspots for Influenza Virus Reassortment. **2013**; 19.

15. Pekoc K. NIH officials assess threat of H5N1: National Institutes of Health **2024**.

16. Schicker RS, Rossow J, Eckel S, et al. Outbreak of Influenza A(H3N2) Variant Virus Infections Among Persons Attending Agricultural Fairs Housing Infected Swine — Michigan and Ohio, July–August 2016. . Morbidity and Mortality Weekly Report **2016**; 65:1157–60.

17. Pigott DM, Millear AI, Earl L, et al. Updates to the zoonotic niche map of Ebola virus disease in Africa. eLife **2016**; 5.

18. Ebola Disease Outbreaks by Species and Size, Since 1976. Available at: <https://www.cdc.gov/ebola/outbreaks/index.html>.

19. Gardini Sanches Palasio R, Marques Moralejo Bermudi P, Luiz De Lima Macedo F, Reis Santana LM, Chiaravalloti-Neto F. Zika, chikungunya and co-occurrence in Brazil: space-time clusters and associated environmental–socioeconomic factors. Scientific Reports **2023**; 13.

20. Mocelin HJS, Catão RC, Freitas PSS, et al. Analysis of the spatial distribution of cases of Zika virus infection and congenital Zika virus syndrome in a state in the southeastern region of Brazil: Sociodemographic factors and implications for public health. International Journal of Gynecology &amp; Obstetrics **2020**; 148:61-9.

21. Liang Y, Dai X. The global incidence and trends of three common flavivirus infections (Dengue, yellow fever, and Zika) from 2011 to 2021. Frontiers in Microbiology **2024**; 15.

22. Zika virus Available at: <https://www.who.int/news-room/fact-sheets/detail/zika-virus>. Accessed January 21 2025.

23. Global Health Security Index. Available at: <https://ghsindex.org/>. Accessed January 10 2024.

24. Analysis of country-level health research capacity for the ESSENCE on Health Research Initiative. In: Fogarty International Center NIoH, ed. Bethesda, Maryland, **2022**.

25. Organization WH. International Health Regulations (2005): States Parties Self-assessment annual reporting tool, second edition. . 2 ed. Geneva, **2021**.

26. Bourner J, Vaillant M, Paddy A, et al. Adaptive Design for Phase II/III Platform Trial of Lassa Fever Therapeutics. Emerging Infectious Diseases **2025**; 31.

27. Salami K, Imbault N, Erlebach A, Urban J, Zoglowek M, Tornieporth NG. A systematic scorecard-based approach to site assessment in preparation for Lassa fever vaccine clinical trials in affected countries. Pilot and Feasibility Studies **2020**; 6.

28. ArboNET. Available at: <https://www.cdc.gov/mosquitoes/php/arbonet/index.html>. 2025.

29. Ong J, Chong C-S, Yap G, et al. Gravitrap deployment for adult Aedes aegypti surveillance and its impact on dengue cases. PLOS Neglected Tropical Diseases **2020**; 14:e0008528.

30. Baba MM, Ahmed A, Jackson SY, Oderinde BS. Cryptic Zika virus infections unmasked from suspected malaria cases in Northeastern Nigeria. PLOS ONE **2023**; 18:e0292350.

31. Madewell Z, Hernandez-Romieu, AC., Wong, JM., et al. Sentinel Enhanced Dengue Surveillance System — Puerto Rico, 2012–2022. Morbidity and Mortality Weekly Report **2024**; 73:1-29.

32. Isere E, Fatiregun A, Ajayi I. An overview of disease surveillance and notification system in Nigeria and the roles of clinicians in disease outbreak prevention and control. Nigerian Medical Journal **2015**; 56:161.

33. Health EiG. SORMAS in Nigeria: Adapting a Fully Integrated Surveillance System to Track COVID-19. Available at: <https://www.exemplars.health/emerging-topics/epidemic-preparedness-and-response/digital-health-tools/sormas-nigeria>. 2025.

34. Rohan H. Beyond Lassa Fever: Systemic and structural barriers to disease detection and response in Sierra Leone. PLOS Neglected Tropical Diseases **2022**; 16:e0010423.

35. Marburg virus disease - Rwanda. In: News DO, ed, **2024**.

36. Sharma V, Kaushik S, Kumar R, Yadav JP, Kaushik S. Emerging trends of Nipah virus: A review. Reviews in Medical Virology **2019**; 29:e2010.

37. Global Influenza Surveillance and Response System (GISRS). Available at: <https://www.who.int/initiatives/global-influenza-surveillance-and-response-system#:~:text=Section%20navigation&text=Global%20influenza%20surveillance%20has%20been,viruses%20and%20other%20respiratory%20pathogens>. 2025.

38. WHO guidance for surveillance during an influenza pandemic. Geneva, **2017**.

39. H5 Bird Flu: Current Situation. Available at: <https://www.cdc.gov/bird-flu/situation-summary/index.html#human-cases>. 2025.

40. U.S. Influenza Surveillance: Purpose and Methods. Available at: <https://www.cdc.gov/fluview/overview/index.html>. 2025.

41. McNamara L, Schafer, IJ., Nolen, LD., et al. . Ebola Surveillance — Guinea, Liberia, and Sierra Leone. Morbidity and Mortality Weekly Report **2016**; 65:35-43.

42. Ratnayake R, Crowe SJ, Jasperse J, et al. Assessment of Community Event–Based Surveillance for Ebola Virus Disease, Sierra Leone, 2015. Emerging Infectious Diseases **2016**; 22:1431-7.

43. Keita M, Lucaccioni H, Ilumbulumbu MK, et al. Evaluation of Early Warning, Alert and Response System for Ebola Virus Disease, Democratic Republic of the Congo, 2018–2020. Emerging Infectious Diseases **2021**; 27:2988-98.

44. Kostkova P, Pinheiro Dos Santos W, Massoni T. ZIKA: improved surveillance and forecast of Zika virus in Brazil. European Journal of Public Health **2019**; 29.

45. Wong JCC, Tay M, Hapuarachchi HC, et al. Case report: Zika surveillance complemented with wastewater and mosquito testing. eBioMedicine **2024**; 101:105020.

46. Low JGH, Ooi EE, Vasudevan SG. Current Status of Dengue Therapeutics Research and Development. The Journal of Infectious Diseases **2017**; 215:S96-S102.

47. Khanam A, Gutiérrez-Barbosa H, Lyke KE, Chua JV. Immune-Mediated Pathogenesis in Dengue Virus Infection. Viruses **2022**; 14:2575.

48. Ogbaini-Emovon E, Akpede G, Okogbenin S, et al. Virus Load Kinetics in Lassa Fever Patients Treated With Ribavirin: A Retrospective Cohort Study From Southern Nigeria. Open Forum Infectious Diseases **2024**; 11.

49. Reyna R, Littlefield K, Shehu N, Makishima T, Maruyama J, Paessler S. The Importance of Lassa Fever and Its Disease Management in West Africa. Viruses **2024**; 16:266.

50. Kortepeter MG, Dierberg K, Shenoy ES, Cieslak TJ. Marburg virus disease: A summary for clinicians. International Journal of Infectious Diseases **2020**; 99:233-42.

51. Johnson K, Vu M, Freiberg AN. Recent advances in combating Nipah virus. Faculty Reviews **2021**; 10.

52. Liang Y. Pathogenicity and virulence of influenza. Virulence **2023**; 14.

53. Jones JC, Yen H-L, Adams P, Armstrong K, Govorkova EA. Influenza antivirals and their role in pandemic preparedness. Antiviral Research **2023**; 210:105499.

54. de La Vega M-A, Caleo G, Audet J, et al. Ebola viral load at diagnosis associates with patient outcome and outbreak evolution. The Journal of Clinical Investigation **2015**; 125:4421-8.

55. Li J, Duan H-J, Chen H-Y, et al. Age and Ebola viral load correlate with mortality and survival time in 288 Ebola virus disease patients. International Journal of Infectious Diseases **2016**; 42:34-9.

56. Halai U-A, Nielsen-Saines K, Moreira ML, et al. Maternal Zika Virus Disease Severity, Virus Load, Prior Dengue Antibodies, and Their Relationship to Birth Outcomes. Clinical Infectious Diseases **2017**; 65:877-83.

57. Bos S, Graber AL, Cardona-Ospina JA, et al. Protection against symptomatic dengue infection by neutralizing antibodies varies by infection history and infecting serotype. Nature Communications **2024**; 15.

58. Gil L, Martín A, Lazo L. Wanted Dead or Alive: A Correlate of Protection Against Dengue Virus. Frontiers in Immunology **2019**; 10.

59. Vannice KS, Wilder-Smith A, Barrett ADT, et al. Clinical development and regulatory points for consideration for second-generation live attenuated dengue vaccines. Vaccine **2018**; 36:3411-7.

60. Oestereich L, Müller-Kräuter H, Pallasch E, Strecker T. Passive Transfer of Animal-Derived Polyclonal Hyperimmune Antibodies Provides Protection of Mice from Lethal Lassa Virus Infection. Viruses **2023**; 15:1436.

61. Ugwu C, Olumade T, Nwakpakpa E, et al. Humoral and cellular immune responses to Lassa fever virus in Lassa fever survivors and their exposed contacts in Southern Nigeria. Scientific Reports **2022**; 12.

62. Murphy H, Ly H. Understanding Immune Responses to Lassa Virus Infection and to Its Candidate Vaccines. Vaccines **2022**; 10:1668.

63. Warner BM, Safronetz D, Stein DR. Current perspectives on vaccines and therapeutics for Lassa Fever. Virology Journal **2024**; 21.

64. Hunegnaw R, Honko AN, Wang L, et al. A single-shot ChAd3-MARV vaccine confers rapid and durable protection against Marburg virus in nonhuman primates. Science Translational Medicine **2022**; 14.

65. Monath TP, Nichols R, Feldmann F, et al. Immunological correlates of protection afforded by PHV02 live, attenuated recombinant vesicular stomatitis virus vector vaccine against Nipah virus disease. Frontiers in Immunology **2023**; 14.

66. Leyva-Grado VH, Promeneur D, Agans KN, et al. Establishing an immune correlate of protection for Nipah virus in nonhuman primates. npj Vaccines **2024**; 9.

67. Krammer F, Jacqueline, Othmar, et al. Meeting Report From “Correlates of Protection for Next Generation Influenza Vaccines: Lessons Learned From the COVID‐19 Pandemic”. Influenza and Other Respiratory Viruses **2024**; 18.

68. Johnson & Johnson Joins World Health Organization in Efforts to Prevent Spread of Ebola in West Africa. New Brunswick, New Jersey Johnson & Johnson **2021**.

69. Puri A, Pollard AJ, Schmidt-Mutter C, et al. Long-Term Clinical Safety of the Ad26.ZEBOV and MVA-BN-Filo Ebola Vaccines: A Prospective, Multi-Country, Observational Study. Vaccines **2024**; 12:210.

70. Warfield KL, Howell KA, Vu H, et al. Role of Antibodies in Protection Against Ebola Virus in Nonhuman Primates Immunized With Three Vaccine Platforms. The Journal of Infectious Diseases **2018**; 218:S553-S64.

71. Parish LA, Stavale EJ, Houchens CR, Wolfe DN. Developing Vaccines to Improve Preparedness for Filovirus Outbreaks: The Perspective of the USA Biomedical Advanced Research and Development Authority (BARDA). Vaccines **2023**; 11:1120.

72. Maciejewski S, Ruckwardt TJ, Morabito KM, et al. Distinct neutralizing antibody correlates of protection among related Zika virus vaccines identify a role for antibody quality. Science Translational Medicine **2020**; 12:eaaw9066.

73. Young G, Bohning KJ, Zahralban-Steele M, et al. Complete Protection in Macaques Conferred by Purified Inactivated Zika Vaccine: Defining a Correlate of Protection. Scientific Reports **2020**; 10.

74. Woodson SE, Morabito KM. Continuing development of vaccines and monoclonal antibodies against Zika virus. npj Vaccines **2024**; 9.

75. Comer JE, Brasel T, Massey S, et al. Natural History of Marburg Virus Infection to Support Medical Countermeasure Development. Viruses **2022**; 14:2291.

76. Alfson KJ, Goez-Gazi Y, Gazi M, et al. Development of a Well-Characterized Cynomolgus Macaque Model of Marburg Virus Disease for Support of Vaccine and Therapy Development. Vaccines **2022**; 10:1314.

77. First FDA-approved vaccine for the prevention of dengue disease in endemic regions. Available at: <https://www.fda.gov/news-events/press-announcements/first-fda-approved-vaccine-prevention-dengue-disease-endemic-regions>.

78. Ongoing | Infectious Disease Accelerated Approvals Vaccines, **2024**.

79. Weir JP, Gruber MF. An overview of the regulation of influenza vaccines in the United States. Influenza and Other Respiratory Viruses **2016**; 10:354-60.

80. Administration USFaD. Guidance for Industry Clinical Data Needed to Support the Licensure of Seasonal Inactivated Influenza Vaccines Available at: <https://www.fda.gov/files/vaccines,%20blood%20&%20biologics/published/Guidance-for-Industry--Clinical-Data-Needed-to-Support-the-Licensure-of-Seasonal-Inactivated-Influenza-Vaccines.pdf>.

81. Guidance for Industry Influenza: Developing Drugs for Treatment and/or Prophylaxis Available at: <https://www.fda.gov/files/drugs/published/Influenza--Developing-Drugs-for-Treatment-and-or-Prophylaxis.pdf>.

82. Malik S, Asghar M, Waheed Y. Outlining recent updates on influenza therapeutics and vaccines: A comprehensive review. Vaccine: X **2024**; 17:100452.

83. Administration FaD. First FDA-approved vaccine for the prevention of Ebola virus disease, marking a critical milestone in public health preparedness and response. Available at: <https://www.fda.gov/news-events/press-announcements/first-fda-approved-vaccine-prevention-ebola-virus-disease-marking-critical-milestone-public-health>.

84. Administration USFaD. FDA Approves First Treatment for Ebola Virus. Available at: <https://www.fda.gov/news-events/press-announcements/fda-approves-first-treatment-ebola-virus>.

85. Taki E, Ghanavati R, Navidifar T, Dashtbin S, Heidary M, Moghadamnia M. Ebanga™: The most recent FDA-approved drug for treating Ebola. Frontiers in Pharmacology **2023**; 14.

86. Bourner J, Salam AP, Jaspard M, et al. The West Africa Lassa fever Consortium pre-positioned protocol for a Phase II/III adaptive, randomised, controlled, platform trial to evaluate multiple Lassa fever therapeutics. Wellcome Open Research **2023**; 8:122.

87. CORE Protocol - A phase 1/2/3 study to evaluate the safety, tolerability, immunogenicity, and efficacy of vaccine candidates against (Filoviruses) virus disease in healthy individuals at risk of (Filovirus) virus disease, **2023**.

88. Longini IM, Yang Y, Fleming TR, et al. A platform trial design for preventive vaccines against Marburg virus and other emerging infectious disease threats. Clinical Trials **2022**; 19:647-54.

89. Solidarity Partners – Platform adaptive randomized trial for new and repurposed Filovirus treatments – Core Trial Protocol.

90. Bolay FK, Grandits G, Lane HC, et al. PREVAIL I Cluster Vaccination Study With rVSVΔG-ZEBOV-GP as Part of a Public Health Response in Liberia. The Journal of Infectious Diseases **2019**; 219:1634-41.

91. Organization WH. WHO Reference Reagent , Anti Dengue, WHO Reference Panel, NIBSC code: 05/248 In: Control NIfBSa, ed, **2016**.

92. Organization WH. WHO International Standard, First WHO International Standard for anti-Lassa fever virus antibodies, NIBSC code: 20/202 In: Control NIfBSa, ed, **2021**.

93. Organization WH. WHO International Standard, First WHO International Standard for antibodies to Marburg virus for binding assays (human serum), NIBSC code: 23/146 In: Control NIfBSa, ed, **2024**.

94. Organization WH. WHO/BS/2023.2458 WHO 1st International Standard for Nipah virus antibody. In: (NSB) NaSfBP, ed, **2023**.

95. Organization WH. WHO International Standard, International Standard for antibody to influenza H5N1 virus, NIBSC code: 07/150 In: Control NIfBSa, ed, **2009**.

96. Organization WH. Influenza Reagent WHO 2nd International Standard for antibody to influenza, H1N1pdm virus, NIBSC code: 10/202 In: Control NIfBSa, ed, **2013**.

97. Organization WH. WHO International Standard, 1st International Standard for Ebola virus (EBOV) antibodies - Sierra Leone Convalescent Plasma Pool, NIBSC code: 15/262 In: Control NIfBSa, ed, **2018**.

98. Mattiuzzo G, Knezevic I, Hassall M, et al. Harmonization of Zika neutralization assays by using the WHO International Standard for anti-Zika virus antibody. npj Vaccines **2019**; 4.

99. Organization WH. WHO International Standard, 1st International Standard for anti-Asian lineage Zika virus antibody (human), NIBSC code: 16/352 In: Control NIfBSa, ed, **2018**.
